# The anti-tubercular activity of simvastatin is mediated by cholesterol-driven autophagy via the AMPK-mTORC1-TFEB axis

**DOI:** 10.1194/jlr.RA120000895

**Published:** 2020-08-26

**Authors:** Natalie Bruiners, Noton K. Dutta, Valentina Guerrini, Hugh Salamon, Ken D. Yamaguchi, Petros C. Karakousis, Maria L. Gennaro

**Affiliations:** 1 Public Health Research Institute, New Jersey Medical School, Rutgers, The State University of New Jersey, Newark, NJ, USA; 2 Center for Tuberculosis Research, Department of Medicine, Johns Hopkins University School of Medicine, Baltimore, MD, USA; 3 Knowledge Synthesis Inc., Berkeley, CA, USA; 4 Department of International Health, Johns Hopkins Bloomberg School of Public Health, Baltimore, MD, USA

**Keywords:** *Mycobacterium tuberculosis*, statins, macrophages/monocytes, lipids, mechanistic target of rapamycin complex 1 regulation, immunology, adenosine 5′-monophosphate-activated protein kinase-mechanistic target of rapamycin complex 1-transcription factor EB axis

## Abstract

The rise of drug-resistant tuberculosis poses a major risk to public health. Statins, which inhibit both cholesterol biosynthesis and protein prenylation branches of the mevalonate pathway, increase anti-tubercular antibiotic efficacy in animal models. However, the underlying molecular mechanisms are unknown. In this study, we used an in vitro macrophage infection model to investigate simvastatin’s anti-tubercular activity by systematically inhibiting each branch of the mevalonate pathway and evaluating the effects of the branch-specific inhibitors on mycobacterial growth. The anti-tubercular activity of simvastatin used at clinically relevant doses specifically targeted the cholesterol biosynthetic branch rather than the prenylation branches of the mevalonate pathway. Using Western blot analysis and AMP/ATP measurements, we found that simvastatin treatment blocked activation of mechanistic target of rapamycin complex 1 (mTORC1), activated AMP-activated protein kinase (AMPK) through increased intracellular AMP:ATP ratios, and favored nuclear translocation of transcription factor EB (TFEB). These mechanisms all induce autophagy, which is anti-mycobacterial. The biological effects of simvastatin on the AMPK-mTORC1-TFEB-autophagy axis were reversed by adding exogenous cholesterol to the cells. Our data demonstrate that the anti-tubercular activity of simvastatin requires inhibiting cholesterol biosynthesis, reveal novel links between cholesterol homeostasis, the AMPK-mTORC1-TFEB axis, and *Mycobacterium tuberculosis* infection control, and uncover new anti-tubercular therapy targets.

As our knowledge of host-pathogen interactions grows for many infectious agents, pharmacologically manipulating the host response has emerged as a key approach to control or treat disease-causing infections. This approach may be best suited for infections caused by intracellular pathogens, given the intimate relationship between these pathogens and their host cells. For example, *Mycobacterium tuberculosis*, the intracellular pathogen causing tuberculosis, can subvert and disable the antimicrobial mechanisms of the host macrophage while adapting to the environmental conditions created by these mechanisms (1, 2). The treatment of active tuberculosis, which typically presents with lung tissue damage, is prolonged and requires multiple drugs, presumably due to the presence of phenotypically diverse *M. tuberculosis* subpopulations that exhibit antibiotic tolerance and poor penetration of antibiotics into infected tissues (3–5). The prolonged duration of anti-tubercular antibiotic therapy (a minimum of six months for drug-susceptible uncomplicated tuberculosis) poses logistical difficulties for treatment providers and leads to poor patient compliance, particularly in low-resource regions (6). A dramatic consequence of these challenges is the development of antibiotic resistance, which requires treatments that are even more prolonged, less effective, more expensive, and more toxic (7).

One strategy that can address the above challenges is utilizing adjunctive chemotherapies that modify host responses to infection to reduce inflammation and tissue damage and/or to promote infection clearance (8). These host-directed therapies may shorten treatment duration, combat antibiotic-resistant disease by helping reduce its incidence, and improve treatment success, because it is unlikely that cross-resistance develops between antibiotics and host-directed therapeutics. The renewed attention to host-directed therapeutics warrants elucidating their mechanism of action in the context of the target infectious disease. Among the current drugs under evaluation as host-directed therapeutics against tuberculosis are statins (9–13). These drugs, which are prescribed worldwide as cholesterol-lowering agents, act by competitively inhibiting HMG-CoA reductase, the rate-limiting enzyme of the mevalonate pathway (14). This pathway regulates biosynthesis of cholesterol and isoprenoids, such as farnesyl pyrophosphate and geranylgeranyl pyrophosphate. The latter molecules are needed for protein prenylation, a process that activates and targets to membranes several protein classes that regulate cell growth, differentiation, and cell function (15). Consequently, statins not only possess cholesterol-lowering activity but also exhibit pleotropic effects, such as tissue remodeling, inhibition of vascular inflammation, cytokine production, and immunomodulation (16, 17).

Due to their pleotropic effects, statins are currently under investigation in many pathological contexts. For example, their use has been explored against multisystem microbial infections, such as sepsis and pneumonia, and in noninfectious pathologies, such as autoimmune and inflammatory diseases (18–20). In regard to tuberculosis, statins exhibit anti-tubercular activity in murine and human macrophages infected ex vivo (9, 12, 13), display immunomodulatory effects in ex vivo-infected human peripheral blood cells (13), and shorten the duration of antibiotic treatment in *M. tuberculosis*-infected mice (10, 11). Previous work (9) found a reduction in *M. tuberculosis* burden in murine macrophages by high doses of statins that inhibited both the cholesterol- and isoprenoid-forming branches of the mevalonate pathway. These effects were ascribed to the induction of autophagy, which controls *M. tuberculosis* infection (21–23), through the inhibition of geranylgeranyl biosynthesis, as observed in coronary arterial myocytes and certain cancer cell lines (24, 25). The molecular mechanisms underlying these results and the significance of effects induced by statin doses that far exceed therapeutic dosing remained unexplained. By utilizing simvastatin at drug concentrations compatible with clinical dosing (C_max_ 1 μM) (26) to treat *M. tuberculosis*-infected human monocytic cells, we now show that the anti-tubercular activity of statins results from their cholesterol-lowering effects and the consequent regulation of the nutrient- and energy-sensing pathways regulated by the AMP-activated protein kinase (AMPK), the mechanistic target of rapamycin complex 1 (mTORC1), and the transcription factor EB (the AMPK-mTORC1-TFEB axis) in ways that promote autophagy and control of infection with intracellular pathogens.

## METHODS

### Antibodies and reagents

Simvastatin hydroxy acid was purchased from Santa Cruz Biotechnology (Dallas, TX). GGTI 298 trifluoroacetate salt hydrate and U18666A were purchased from Cayman Chemical Co. (Ann Arbor, MI). FTI-277 trifluoroacetate salt, BM15766 sulfate, water-soluble cholesterol-methyl-β-cyclodextrin, and l-arginine were obtained from Sigma-Aldrich (St. Louis, MO). Digoxin, dorsomorphin, and everolimus were purchased from SelleckChem (Houston, TX). The cholesterol quantification kit was purchased from BioVision Inc. (Milpitas, CA) and was performed according to the manufacturer’s protocol. Antibodies against mTOR (catalog #2983), phospho-mTOR (Ser2448, catalog #2971), tfeb (catalog #32361), sequestosome 1 (SQSTM1/p62, catalog #88588), β-actin (catalog #4970) and lamin B1 (catalog #12586) were purchased from Cell Signaling Technology (Danvers, MA). Antibodies against Rab escort protein 1 (REP-1; catalog #sc-23905), Ras homolog family member B (RhoB; catalog #sc-180), Ras-related protein Rap-1A (catalog #sc-14872-R, specific for unprenylated Rap1a), and Ras-related protein Rab5 (catalog #sc-46692), were purchased from Santa Cruz Biotechnology. Secondary antibodies IRDye® 800CW goat anti-rabbit IgG, IRDye® 680RD donkey anti-mouse IgG, and IRDye® 800CW goat anti-mouse IgG were purchased from LI-COR Biosciences (Lincoln, NE).

### Culturing of cell lines

THP1 monocytes were purchased from ATCC (Manassas, VA). THPI monocytes were grown in RPMI 1640 culture medium (Corning, Manassas, VA) supplemented with 4 mM l-glutamine (Corning), 10% FBS (Seradigm, Radnor, PA), and penicillin-streptomycin solution (Corning) incubated at 37°C in a humidified atmosphere consisting of 5% CO_2_. For differentiation, PMA (Sigma-Aldrich) was added overnight at a final concentration of 40 nM.

### siRNA transfection

Differentiated THP1 cells, seeded at a density of 3.3 × 10^5^ cells/well in 6-well plates were in RPMI-1640 supplemented with 2% FBS (Seradigm) and 4 mM l-glutamine (Corning). Commercially available ON-TARGET plus Nontargeting and CHM SMARTpool siRNA (Dharmacon, Lafayette, CO) was reconstituted with 1× siRNA buffer (Dharmacon) to a stock concentration of 20 μM. Transfection with siRNA was performed at a final concentration of 50 nM using DharmaFECT2 transfection reagent (Dharmacon) according to the manufacturer’s protocol. After 72 h, cells were subjected to a second round of transfection with an identical concentration (50 nM) of siRNA. The second round of siRNA treatment was performed to prolong the duration of silencing the target protein at day 6 of sample collection. After 6 days, the cells were assayed for protein expression by Western blot analysis, as described below.

### In vitro infection of differentiated THP1

Differentiated THP1 cells, seeded at a density of 5 × 10^5^ cells/well in 24-well plates were infected with a frozen stock of *M. tuberculosis* H37Rv at a multiplicity of infection of 1:20, as previously described (11, 27). The bacterial inoculum for infection was prepared by diluting a frozen bacterial stock in supplemented RPMI-1640 medium (as above) to obtain the desired multiplicity of infection. Bacterial clumps were disrupted by vortexing with sterile 3 mm-diameter glass beads for 2 min. The resulting suspension was used for infection of primary and culture cells. Infected cells were washed three times, at 4 h postinfection, to remove extracellular bacteria and were incubated with fresh RPMI-1640 supplemented with 2% FBS (Seradigm) and 4 mM l-glutamine (Corning) and treated with either chemical inhibitors or solvent controls. Medium was replenished at day 3 postinfection. At day 6 postinfection, cells were lysed with 0.05% SDS in water and serial dilutions plated on 7H10 agar plates. After 3 weeks, colony-forming units (CFUs) were enumerated. Cell viability and total number of cells were determined using trypan blue counting.

### Preparation of whole-cell lysate and subcellular fractions

THP1 cells seeded in 6-well plates at a density of 2 × 10^6^ cells/well were treated with compounds for the duration of the experiment. A total of 2 × 10^6^ macrophages were lysed in 100 μl RIPA lysis buffer (Santa Cruz Biotechnology). A subcellular protein fractionation kit for cultured cells (Thermo Fischer Scientific, Waltham, MA) was used to prepare the various subcellular protein extracts according to the manufacturer’s instructions. Protein lysates were filtered and sterilized through a 0.2 μm filter and stored at −20°C.

### Western blot analysis

We used a BCA protein assay (Thermo Fischer Scientific) to determine the protein concentration. Equal amounts of protein were then resolved by SDS-PAGE on either 7.5% or 10% polyacrylamide gels (Bio-Rad, Hercules, CA). Separated proteins were transferred to PVDF Immobilon-FL membranes (Millipore, Billerica, MA) using the Bio-Rad Trans-Blot Turbo transfer system (Hercules, CA). The membrane was blocked with Odyssey Block buffer (LI-COR Biosciences) at RT for 2 h and incubated with primary antibodies (1:1,000) at 4°C overnight. The membrane was then incubated with secondary near-infrared fluorescent antibodies (1:10,000) at RT for 1 h. Immunoblots were scanned using a LI-COR Odyssey infrared imager. Antibody signals were quantified using LI-COR Odyssey infrared imager software version 1.2. Quantitated protein levels were normalized to total protein levels using REVERT total protein stain (LI-COR Biosciences), β-actin, or lamin B1. Immunoblots were stripped with NewBlot PVDF stripping buffer (LI-COR Biosciences) and reprobed when required.

### AMP/ATP assay

Cellular AMP and ATP were extracted using the boiling water method (28). Cells were seeded onto 12-well plates and treated with simvastatin for 6 days. Cells were washed twice with cold PBS, followed by the addition of ice-cold water. Cells were scraped and collected into a prechilled tube and were lysed by vigorous vortexing on ice for 10 s. The protein concentration was determined by the BCA protein assay (Thermo Fischer Scientific) using 10 μl of the lysate. The remaining lysate was boiled with shaking for 10 min, cooled on ice for 30 s, and centrifuged at 15,800 *g* for 5 min. The supernatant was collected and stored at −80°C until further use. The levels of ATP, ADP, and AMP were determined using an ATP/ADP/AMP assay kit (Biomedical Research Service Center, University at Buffalo, State University of New York). The luciferase bioluminescence was measured using the BioTek Synergy H1 microplate reader and was compared with a standard curve of ATP concentrations. AMP concentration was calculated according to the manufacturer’s instructions.

### Statistical analysis

All values are presented as mean ± SD (n = 3). Comparisons between two groups were performed using a two-tailed Student’s *t*-test or one-way ANOVA test for trend. The criterion for statistical significance was *P* < 0.05.

### Transcriptome data analysis

Statistical analysis was performed in R (https://www.r-project.org/) using the Knowledge Synthesis KS/prot data integration and discovery platform (Knowledge Synthesis Inc., Berkeley, CA; knowledgesynthesis.com). To compare measurements of transcript levels between sample groups, *t*-tests were performed, and the resulting *P*-values were calculated. To explore the association between simvastatin and specific protein functions and pathways, an experimental compendium of gene-set analyses of tuberculosis-relevant experiments, including simvastatin-treated macrophages, was established in a searchable results repository, iDataMed (TB version; https://idatamed.com/tb). The compendium was comprised of results calculated in the same manner as in previous research (27, 29, 30) and included analyses of different types of gene sets defined by NCBI biosystems (https://www.ncbi.nlm.nih.gov/biosystems), Reactome (http://reactome.org/), and transcriptional modulators (31). Each gene set was tested for extreme ranks of differential expression among all measured genes in each comparison by coincident extreme ranks in numerical observations. Multiple transcript measurements were combined as described previously (30, 32). The Benjamini-Hochberg method was used to calculate the false discovery rate (FDR) within each gene set type. Search was facilitated in iDataMed/tb by a text query interface, in which searches such as “simvastatin AND mTOR” or “simvastatin AND ribosome biogenesis” returned statistically significant findings sorted in a fashion weighted to present top-ranking and more significant results first.

## RESULTS

### Simvastatin limits *M. tuberculosis* infection by inhibiting de novo synthesis of cholesterol

**Fig. 1.**
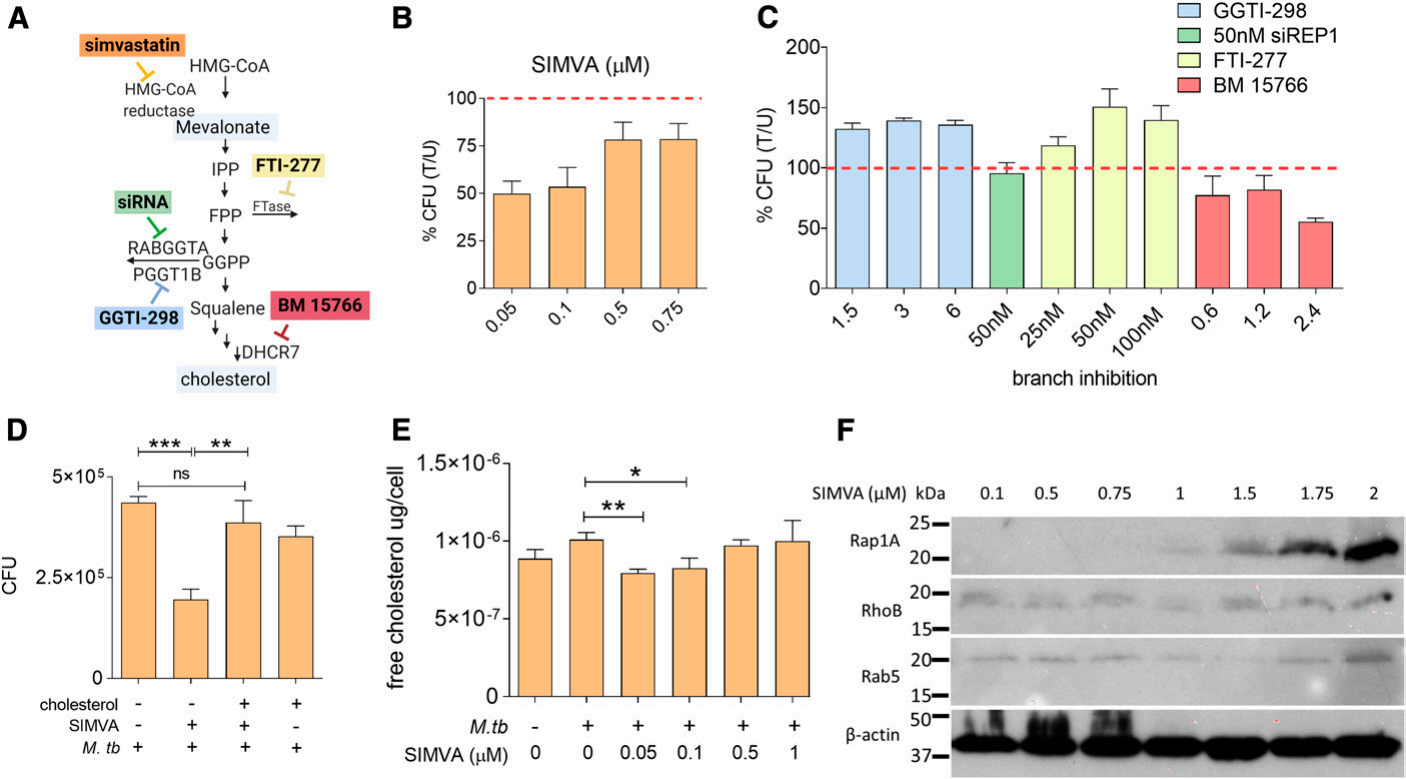
Simvastatin reduces the intracellular burden of *M. tuberculosis* by inhibiting cellular cholesterol biosynthesis. A: Diagram of the mevalonate pathway. IPP, isopentenyl pyrophosphate; FPP, farnesyl pyrophosphate; FTase, farnesyltransferase; GGPP, geranylgeranyl pyrophosphate; RABGGTA, Rab geranylgeranyltransferase subunit alpha; PGGT1B, protein geranylgeranyltransferase type I subunit beta; DHCR7, 7-dehydrocholesterol reductase. The colored boxes represent pharmacological inhibitors of specific enzymes in the pathway (simvastatin, FTI-277, GGTI-298, BM 15766), as indicated. siRNA targeting REP-1 (a chaperone protein that presents Rab proteins for prenylation by GGTase-II) was used because no pharmacological inhibitor of GGTI type 2 was commercially available. B, C: THP1 cells were infected with *M. tuberculosis* H37Rv, followed by treatment with multiple doses of simvastatin (B) or branch inhibitors of the mevalonate pathway (C). CFUs were enumerated at 6 days post treatment. Data are presented as percent CFU changes relative to solvent control. U, solvent-treated; T, treated. The red dashed line represents *M. tuberculosis* CFU levels in the absence of any pharmacological treatment. D: Analysis of cellular cholesterol levels in infected THP1 cells treated with solvent and increasing doses of simvastatin. (E) Intracellular growth of *M. tuberculosis* in THP1 cells treated for 6 days with 100 nM simvastatin in the absence and presence of water-soluble cholesterol (1.25 μg/ml). Significance was tested by Student’s *t*-test: *P < 0.05; **P < 0.01 and ***P < 0.001. F: Western blot analysis of sentinel protein targets of the mevalonate pathway of infected THP1 cells treated with simvastatin (SIMVA; 0.1–2 μM) for 6 days. β-Actin was used as loading control. It is noted that, in particular, the antibody against Rap1A is directed against the unprenylated Rap1A form, and therefore, the signal increases with the simvastatin dose.

The mevalonate pathway, which is the mechanistic target of statins, is multi-branched (**Fig. 1A**). Identifying the pathway branch(es) associated with the observed anti-tubercular activity of these compounds is the first step toward elucidating the underlying mechanism of action. Treatment of *M. tuberculosis*-infected THP1 cells (a human monocytic cell line) with simvastatin demonstrated that this drug decreased *M. tuberculosis* intracellular burden (Fig. 1 B), consistent with previous reports (11, 12). The anti-mycobacterial effect of simvastatin varied with the dose, with concentrations of 50–100 nM being the most effective (Fig. 1B). Additional experiments determined that these anti-mycobacterial doses were nontoxic for THP1 cells (supplemental Fig. S1A) and that simvastatin had no direct antimicrobial activity against *M. tuberculosis* in axenic cultures (supplemental Fig. S1B). We next tested the effect on intracellular *M. tuberculosis* burden of inhibitors specifically targeting each branch of the mevalonate pathway. As shown in Fig. 1A, the mevalonate pathway branches at farnesyl pyrophosphate synthesis, which is used for protein prenylation, including farnesylation and geranylgeranylation, or is converted to squalene, which is required for de novo cholesterol synthesis. We treated *M. tuberculosis*-infected THP1 cells with inhibitors of farnesyl transferase (FTI-277), geranylgeranyl transferase type 1 (GGTI-298) and 7-dehydrocholesterol reductase (BM 15766 sulfate) (colored boxes in Fig. 1A). Moreover, because no inhibitor of geranylgeranyl transferase type 2 (GGTase-II) is commercially available, we used siRNA against the gene encoding the REP-1, a chaperone protein that presents Rab proteins for prenylation by GGTase-II. We found that only cells treated with the cholesterol-branch inhibitor BM 15766, but not other branch-specific inhibitors, showed a reduced mycobacterial burden (35% inhibition relative to solvent-treated cells) (Fig. 1C). In addition, the inhibition of intracellular *M. tuberculosis* growth by simvastatin was reversed by exogenous addition of water-soluble cholesterol (Fig. 1D). We verified the link between simvastatin anti-mycobacterial activity and blockage of the cholesterol biosynthetic branch of the mevalonate pathway, as treatment with *M. tuberculosis*-inhibiting doses of simvastatin (50–100 nM) (Fig. 1 B) reduced the amount of free cholesterol in THP1 cells (Fig. 1E) while inducing no detectable alteration of the cellular ability to prenylate proteins (sentinel targets of each prenylation pathway are shown in Fig. 1F). Taken together, these results clearly demonstrate that the anti-tubercular activity of simvastatin used at 50–100 nM specifically targets the cholesterol biosynthetic branch rather than the prenylation branches of the mevalonate pathway. The loss of anti-tubercular effect observed with higher simvastatin doses may result from altering protein prenylation, which has pleiotropic effects on cellular functions, and/or the decreased ability of macrophages to control infection at toxic or near-toxic drug doses.

### Simvastatin inhibits *M. tuberculosis* growth by inducing autophagy in a cholesterol-dependent manner

Seminal findings by Segal and Bloch (33), which demonstrated that *M. tuberculosis* preferentially utilizes lipids as a carbon and energy source during infection, have spurred vast research efforts into host lipid requirements for survival of this pathogen during infection (34–36). In particular, it is known that *M. tuberculosis* metabolizes cholesterol during infection and that degradation of this sterol contributes to the pathogen’s survival in host cells (37, 38). Therefore, we asked whether the reduction of intracellular *M. tuberculosis* burden by simvastatin was due to the reduced availability of host cholesterol as a nutrient source during host cell infection. Catabolism of cholesterol by β oxidation produces propionyl-CoA, which is assimilated into pyruvate via the methyl citrate cycle (39). *M. tuberculosis* mutants that are defective in methyl citrate cycle enzymes cannot grow either in synthetic media containing cholesterol as a primary carbon source or in macrophages (36). Moreover, the intracellular growth defect of such mutants is relieved by additional mutations impairing mycobacterial cholesterol uptake (39), implying that accumulation of propionyl-CoA is toxic. Therefore, we predicted that pharmacologically induced reduction of host cell cholesterol should relieve the intracellular growth defect of methyl citrate cycle*-*deficient *M. tuberculosis* mutants.

**Fig. 2.**
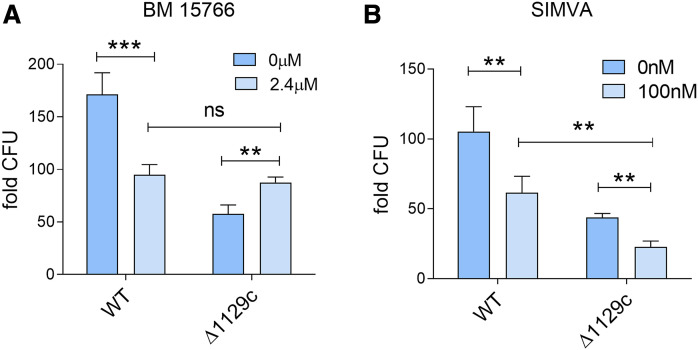
Reduction of *M. tuberculosis* growth by simvastatin is independent of mycobacterial utilization of cholesterol as a carbon source. A, B: THP1 cells were infected with WT or deletion mutant (ΔRv1129c) *M. tuberculosis* strains for 6 days and treated with either BM 15766 (inhibitor of 7-dehydrocholesterol reductase) (2.4 μM) (A) or simvastatin (100 nM) (B). At day 6 posttreatment, cells were lysed, plated on Middlebrook 7H10 agar plates, and incubated for 3 weeks at 37°C for bacterial CFU enumeration. Data represent fold change in CFU at 6 days relative to 4 h postinfection. Significance was tested by Student’s *t*-test: **P < 0.01 and ***P < 0.001.

To test our hypothesis, we used *M. tuberculosis* WT and a mutant strain genetically inactivated in *rv1129c,* which encodes a transcriptional factor required to induce the methyl citrate cycle genes (39, 40). When we infected THP1 cells with *M. tuberculosis* WT and Δ*rv1129c* mutant strains, we found that the mutant strain was attenuated for growth in macrophages, as expected (up to 3-fold reduction of CFUs relative to WT) (**Fig. 2A**, dark blue bars). Treating infected cells with BM 15766 sulfate (which specifically inhibits de novo cholesterol synthesis, Fig. 1A) drastically reduced intracellular growth of the WT strain (45%), as expected, but increased growth in the Δ*rv1129c* mutant strain by 50% (Fig. 2A). As a result, the infection burden of WT and Δ*rv1129c* mutant strains was essentially indistinguishable in drug-treated cells (Fig. 2A), confirming our hypothesis that the pharmacological reduction of intracellular host cholesterol as nutrient source relieves the Δ*rv1129c* mutant strain from cholesterol toxicity and greatly alleviates the mutant growth defect. In contrast, simvastatin treatment inhibited both WT and mutant growth to a similar extent (>40% growth inhibition) and did not relieve the growth defect of the mutant strain relative to WT (Fig. 2B). The different effect of the two drugs on mutant growth strongly implies that the reduced intracellular burden of *M. tuberculosis* in simvastatin-treated cells is not primarily (or solely) caused by the reduced availability of cholesterol as carbon source for mycobacterial growth.

**Fig. 3.**
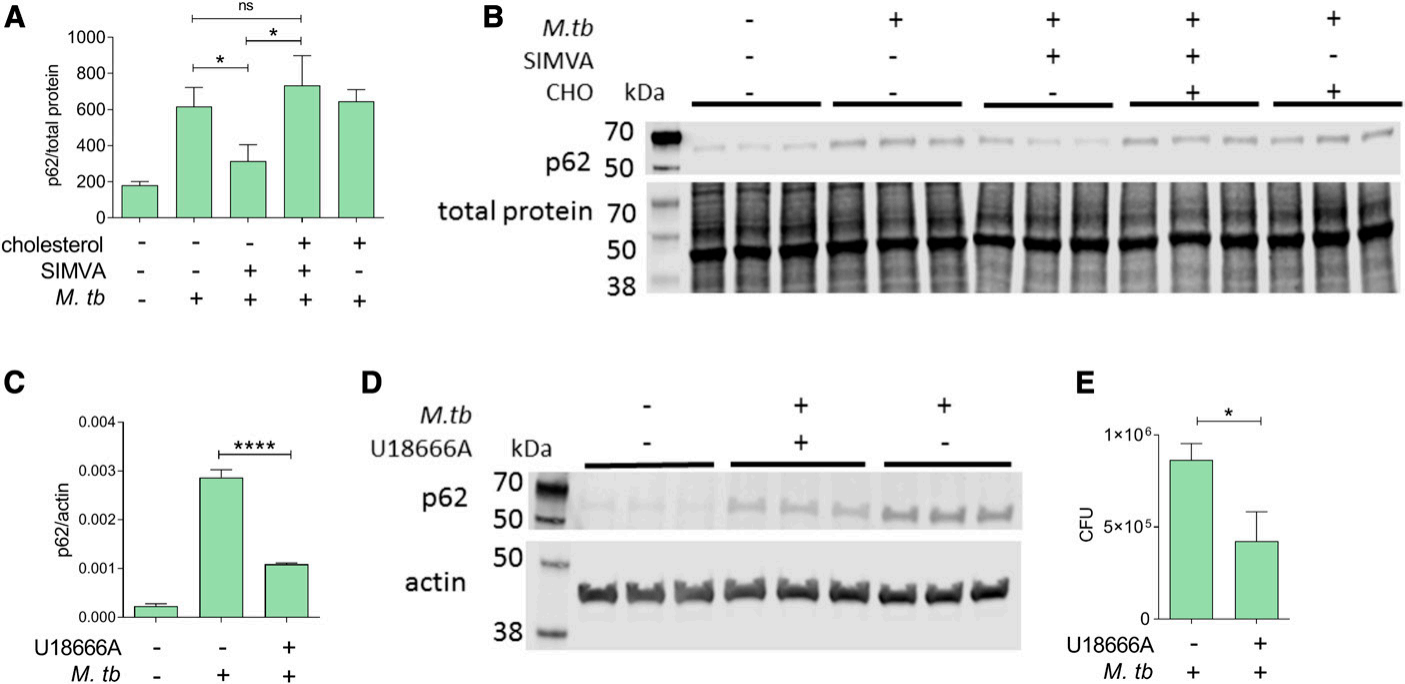
Reduction of *M. tuberculosis* growth by simvastatin is associated with cholesterol-dependent induction of autophagy. A, C: Immunoblot analysis of the abundance of p62/SQSTM1 and β-actin or total protein in whole-cell lysates obtained from THP1 cells infected with *M. tuberculosis* for 6 days and treated with DMSO as a solvent control or 100 nM simvastatin in the absence or presence of water-soluble cholesterol (1.25 μg/ml) (A) and U186666A (inhibitor of cholesterol transport and synthesis) (1.25 μM) (C). Protein quantification and normalization relative to total protein or β-actin per lane was performed using LI-COR Image Studio software. B, D: Representative immunoblots of p62/SQSTM1 in whole-cell lysates of THP1 cells treated with 100 nM simvastatin in the presence of soluble cholesterol (1.25μg/ml) (B) and U186666A (1.25 μM) (D). When total protein was used as loading control (panel B), only a portion of the membrane probed for total protein is shown. E: Effect of U18666A on intracellular growth of *M. tuberculosis* in THP1 cells. Significance was tested by Student’s *t*-test: *P < 0.05 and ****P < 0.0001; ns, not significant.

A previous report showed that simvastatin induces autophagy, a known anti-mycobacterial function (41), in *M. tuberculosis*-infected bone marrow-derived macrophages (9). That effect was observed with high drug doses (50 μM), well above those affecting all branches of the mevalonate pathway (Fig. 1F) and that are toxic to THP1 cells (supplemental Fig. S1A). We used simvastatin at 100 nM concentration to examine whether this drug induces autophagy at a dose that is anti-mycobacterial (Fig. 1B) while only affecting cholesterol biosynthesis (but not protein prenylation) in THP1 cells (Fig. 1E, F). To assess the effects of simvastatin treatment on autophagy, we measured the levels of the autophagy marker p62/sequestosome 1, which is reduced upon induction of autophagy and increased when autophagy is impaired (42). As expected, infection of THP1 cells with *M. tuberculosis* increased p62 levels (**Fig. 3A**, B) because *M. tuberculosis* infection inhibits autophagy (43). When we treated the infected cells with simvastatin, we found that p62 protein levels decreased (Fig. 3A, B), indicating activation of autophagy. The effect of simvastatin on p62 protein levels was reversed by the addition of exogenous cholesterol, demonstrating a role for cholesterol in autophagy regulation by statins (Fig. 3A, B).

If interfering with cholesterol homeostasis modulates autophagy, treating infected macrophages with a class of drugs (other than statins) impacting cellar cholesterol homeostasis should also induce autophagy and have anti-mycobacterial activity. When we treated *M. tuberculosis*-infected THP1 cells with U18666A, which inhibits intracellular cholesterol transport (44), we observed a >60% reduction of p62 levels compared with infected solvent-treated control cells (Fig. 3C, D). The autophagy effect was accompanied by anti-mycobacterial activity (>45% *M. tuberculosis* growth inhibition, Fig. 3E). Taken together, these results demonstrate that simvastatin induces autophagy by altering cellular cholesterol homeostasis.

### Simvastatin induces autophagy by inhibiting mTORC1 activation in a cholesterol-dependent manner

**Fig. 4.**
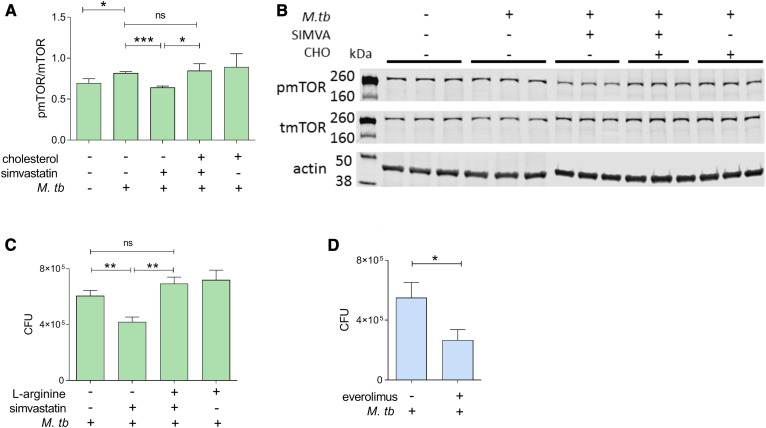
Reduction of *M. tuberculosis* growth by simvastatin is associated with cholesterol-dependent reduction of mTORC1 activation. A: Immunoblot analysis of the phosphorylated/total mTORC1 ratio normalized to β-actin in whole-cell lysates of uninfected and infected THP1 cells for 6 days treated with DMSO as a solvent control or 100 nM simvastatin in the absence and presence of soluble cholesterol (1.25 μg/ml). Protein quantification and normalization relative to β-actin per lane was performed using LI-COR Image Studio software. B: A representative image of immunoblots as shown in A. C: Intracellular growth of *M. tuberculosis* in THP1 cells infected for 6 days and treated with 100 nM simvastatin in the absence or presence of l-arginine (0.78 mM). D: Effect of the mTORC1 inhibitor everolimus on intracellular growth of *M. tuberculosis* in THP1 cells. Significance was tested by Student’s *t*-test: *P < 0.05, **P < 0.01 and ***P < 0.001; ns, not significant.

A key inhibitor of autophagy is mTORC1, a serine-threonine protein kinase that functions as a nutrient/energy/redox sensor. Statins can alter mTORC1 signaling and autophagy in coronary arterial myocytes by affecting prenylation-dependent mechanisms (24). Therefore, we tested to determine whether simvastatin treatment at 100 nM, which only affects the cholesterol biosynthetic branch of the mevalonate pathway (Fig. 1C, F), inhibited mTORC1 activity in *M. tuberculosis*-infected cells. Because the enzymatic activity of mTORC1 is dependent on phosphorylation of the mTOR subunit, we measured phosphorylation at serine 2448, which is an mTORC1 activation marker (45–47), in infected THP1 cells treated with solvent control or 100 nM simvastatin. We found that *M. tuberculosis* infection increased mTORC1 activation, while simvastatin decreased it to uninfected levels (**Fig. 4A**, B). Further, simvastatin-mediated reduction of mTORC1 activation was reversed by supplementing the cell culture with water-soluble cholesterol (Fig. 4A, B). This result links the simvastatin effects on mTORC1 activity to cellular cholesterol homeostasis. To further establish the relationship between mTORC1 activity and simvastatin-mediated induction of autophagy, we supplemented simvastatin-treated cells with l-arginine, a known activator of mTORC1 (48). We found that adding l-arginine abolished the simvastatin-induced inhibition of *M. tuberculosis* burden (Fig. 4C), implying that mTORC1 affects mycobacterial burden. Indeed, treatment with everolimus, an allosteric inhibitor of the mTOR subunit of the mTORC1 complex (49, 50), reduced the *M. tuberculosis* burden of THP1 cells by 25% (Fig. 4D). Taken together, these results indicate that reduction of cellular cholesterol inhibits mTORC1 activity, thereby inducing autophagy and, consequently, inhibiting *M. tuberculosis* infection.

### Simvastatin regulates the nuclear translocation of TFEB

**Fig. 5.**
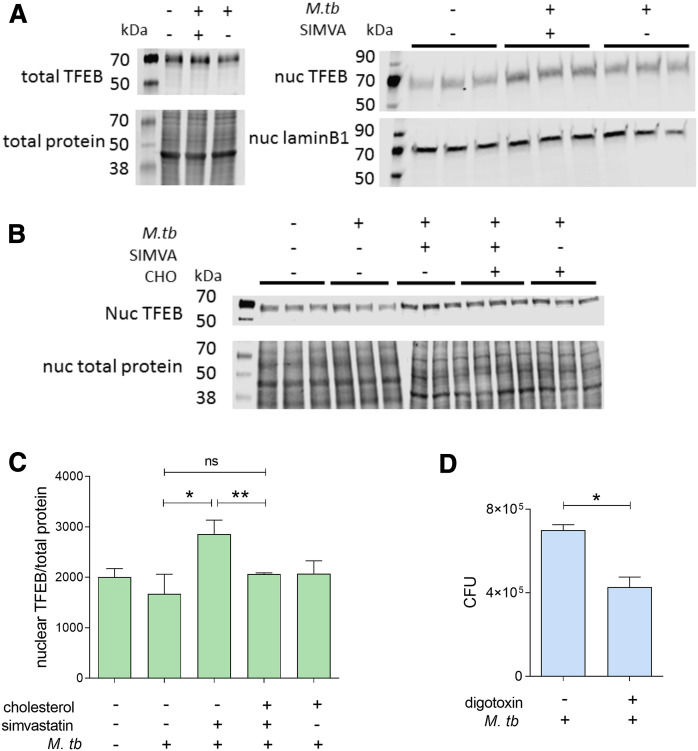
Reduced *M. tuberculosis* burden by simvastatin is associated with cholesterol-dependent nuclear translocation of TFEB. A: Representative immunoblots of TFEB in whole-cell lysates (left panel) and nuclear extracts (right panel) of THP1 cells uninfected or infected with *M. tuberculosis* for 6 days treated with DMSO as a solvent control or 100 nM simvastatin. Protein quantification and normalization relative to total protein or lamin B1, per lane, was performed using LI-COR Image Studio software. B: Immunoblot analysis of TFEB abundance (relative to total protein) in nuclear lysates of infected THP1 cells treated with 100 nM simvastatin in the absence or presence of soluble cholesterol (1.25 μg/ml). When total protein was used as loading control (panels A and B), only a portion of the membrane probed for total protein is shown. C: Effect of 7.5 nM Digoxin (TFEB activator) on the intracellular growth of *M. tuberculosis* in THP1 cells. Significance was tested by Student’s *t*-test: *P < 0.05; **P < 0.01; ns, not significant.

We next sought to identify the mTORC1-dependent pathway(s) through which simvastatin induces autophagy and controls *M. tuberculosis* infection. A prime candidate is TFEB, a transcription factor involved in lysosomal biogenesis and autophagy induction (51, 52). mTORC1 inhibits the nuclear translocation of TFEB, which is required for its activation (53, 54). We found that simvastatin treatment increased TFEB abundance in the nuclear fraction but not in the total extracts obtained from *M. tuberculosis*-infected cells compared with infected control cells (exposed to solvent alone) (**Fig. 5A**). Exogenously added cholesterol reversed the simvastatin effect on TFEB nuclear localization (Fig. 5B). Moreover, treatment with digoxin, a TFEB activator, reduced the intracellular bacillary burden by 30% in infected THP1 cells (Fig. 5C). Taken together, these data imply that by controlling cholesterol levels, simvastatin induces nuclear localization and consequent activation of TFEB, an mTORC1-regulated transcription factor that induces autophagy.

### Simvastatin regulates transcription of the mTORC1 and TFEB pathways

We next sought corroboration of our THP1 mechanistic results in published transcriptomics data generated with simvastatin-treated human cells. We searched for relevant molecular mechanisms by reanalyzing the results of a gene expression study of simvastatin-treated human peripheral blood mononuclear cells (PBMCs) performed in the context of cardiovascular disease [GSE4883 and (55)]. We found strong evidence that TFEB-regulated genes were upregulated by simvastatin treatment of PBMCs (TFEB-bound in **Table 1**). Moreover, the mTOR signaling pathway was also upregulated (Table 1). The top three drivers of this result were genes encoding growth factor receptor-bound protein 10 (GRB10), unc-51 like autophagy-activating kinase 2 (ULK2), and sestrin 2 (SESN2) (supplemental Table S1). Upregulation of these three genes is associated with diminished mTORC1 activity because GRB10 and SESN2 are negative regulators of the mTORC1 signaling pathway (56, 57), while ULK2, which promotes autophagy (58), is downregulated by mTORC1 (59). In addition, pathways expressing functions that require mTORC1 activation, such as ribosome biogenesis and hypoxia factor 1α (HIF1A)-bound genes [HIF1A activity is induced by mTORC1 (60)], were reduced by simvastatin treatment, while insulin-receptor signaling, which is inhibited by mTORC1 (61), was found upregulated in simvastatin-treated cells. Thus, simvastatin treatment induced a clear transcriptomic signature of decreased mTORC1 activity and increased autophagy in human primary cells (Table 1). These data corroborate our mechanistic results with THP1 cells.

**TABLE 1.** Transcriptomic changes of signaling pathways associated with mTORC1 and TFEB in simvastatin-treated PBMCs (GEO ID: GSE4883)

Pathway	Rank	*P*	FDR	Gene-Set Source
↑ TFEB bound genes	3	0.00038	0.00617	ChIP
↑ mTOR signaling	13	5.82E-05	0.0047	KEGG Pathways
↑ IRS activation	17	0.00011	0.00723	Reactome Pathways
↓ Ribosome biogenesis	21	8.34E-06	0.00034	KEGG Pathways
↓ HIF1A bound genes	29	0.00053	0.0013	ChIP
↑ Autophagy	61	0.00159	0.01609	KEGG Pathways

Shown are pathways of interest ranked according to increased or decreased transcriptional changes that are statistically significant.

### Simvastatin induces activation of AMPK

**Fig. 6.**
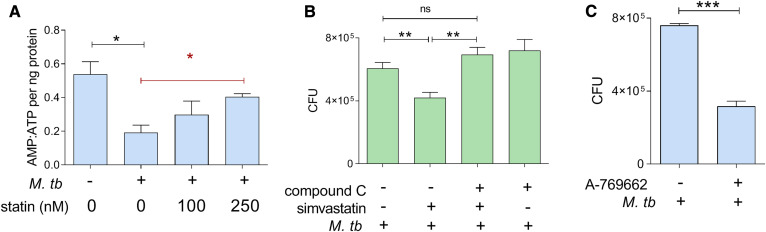
Reduction of *M. tuberculosis* growth by simvastatin is associated with regulation of the AMPK pathway. A: AMP:ATP ratio in THP1 cells treated with 100 and 250 nM simvastatin, which are both anti-tubercular (supplemental Fig. S3). statin, simvastatin. B: Intracellular growth of *M. tuberculosis* in THP1 cells infected for 6 days and treated with DMSO as a solvent control or 100 nM simvastatin in the absence or presence of compound C (AMPK inhibitor) (110 nM). C: Effect of 25 μM A-769662 (AMPK activator) on the intracellular growth of M. *tuberculosis* in THP1 cells. Significance was tested by Student’s *t*-test (black lines) or by one-way ANOVA test for trend (red line): *P < 0.05; **P < 0.01; ***P < 0.001; ns, not significant.

Our pathway analysis of simvastatin-treated PBMCs also identified upregulation of signaling pathways regulated by AMPK, a serine-threonine kinase that inhibits mTORC1 signaling and induces autophagy (62), and upregulated liver kinase B1 (LKB1), a serine-threonine kinase that activates AMPK (63) (**Table 2**), as previously observed in squamous cell carcinoma (64). Because these two proteins participate in the energy-sensing cascade activated by an increased AMP:ATP ratio (63), we first asked whether simvastatin treatment alters cellular AMP:ATP ratios in *M. tuberculosis*-infected cells. We did find that simvastatin reversed the infection-induced decrease in the AMP:ATP ratio (**Fig. 6A**). In accord with these results, treating THP1 cells with compound C, an AMPK inhibitor (65), reversed the reduction of intracellular *M. tuberculosis* burden by simvastatin (Fig. 6B). Furthermore, treatment of infected THP1 cells with A-769662, an AMPK activator (66), reduced *M. tuberculosis* burden by 40% (Fig. 6C). Thus, simvastatin favors the anti-tubercular effect of AMPK by positively regulating both AMPK activation state through AMP:ATP levels and AMPK expression levels.

**TABLE 2.** Transcriptomic changes of signaling pathways associated with AMPK activation in simvastatin-treated PBMCs (GEO ID: GSE4883)

Pathway	Rank	*P*	FDR	Gene-Set Source
↑ LKB1 signaling	3	5.65E-06	0.00343	Pathway Interaction Database
↑ AMPK signaling	69	0.0036	0.03063	KEGG Pathways

Shown are pathways of interest ranked according to increased or decreased transcriptional changes that are statistically significant.

## DISCUSSION

**Fig. 7.**
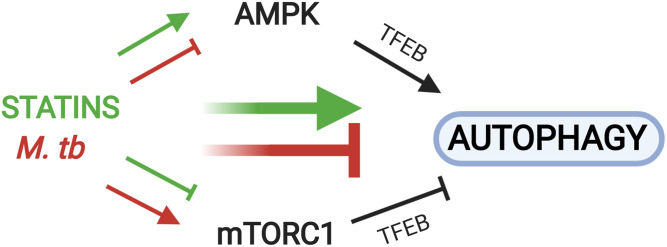
Statins and *M. tuberculosis* infection exhibit opposing effects on AMPK, mTORC1, and autophagy. Reduction of cellular cholesterol content by simvastatin (green arrows) inhibits mTORC1 activation and induces AMPK activation, both of which lead to increased nuclear translocation of TFEB to induce the expression of autophagy-related genes. *M. tuberculosis* infection (red arrows) induces opposing effects, as it blocks AMPK and induces mTORC1 activation to prevent nuclear translocation of TFEB and block autophagy. For simplicity, the effects of AMPK on mTORC1 activation status and vice versa are not shown.

Here, we show that inhibition of the cholesterol biosynthesis branch of the mevalonate pathway underlies the anti-tubercular activity of statins. Simvastatin reduces *M. tuberculosis* burden in human macrophages by decreasing cholesterol levels, thereby regulating the AMPK-mTORC1-TFEB axis in ways that promote autophagy, which is anti-mycobacterial. The anti-tubercular effects of statin-mediated cholesterol reduction occur by inducing autophagy rather than by limiting access to cholesterol as a carbon source for intracellular mycobacteria. Moreover, statin treatment of human cells has transcriptional effects on mTORC1 and AMPK signaling pathways. Together, these findings reveal the hitherto unknown mechanistic foundations of the anti-tubercular activity of statins (diagrammed in **Fig. 7**). Our results support the ongoing testing of statins as adjunctive therapy against tuberculosis (67) and identify novel cellular targets for additional host-directed anti-tubercular treatments.

Our findings fully agree with the known mechanistic links between cellular cholesterol homeostasis and mTORC1 signaling. The lysosome is the cellular site for mTORC1 activation (60) as well as a major sorting site for cellular cholesterol through the Nieman-Pick C (NPC) multi-protein machinery (68). Cholesterol homeostasis is critical for activating mTORC1, because depleting cellular cholesterol with methyl-β-cyclodextrin (69) and blocking cholesterol egress from the lysosome with itraconazole (70) or by siRNA-mediated knockdown of the NPC genes (70) all suppress mTORC1 signaling. We find that statins inhibit mTORC1 signaling at cholesterol-lowering doses and in a cholesterol-dependent manner. Moreover, the compound U18666A, which inhibits the NPC machinery (71), phenocopies the statin effects on autophagy and *M. tuberculosis* burden. Thus, mTORC1 senses cholesterol content and cholesterol trafficking inside the cell, while statins inhibit mTORC1 signaling by dysregulating cholesterol homeostasis. It is worth noting that, while our work and the above-cited literature strongly link mTORC1 inhibition by statins with their cholesterol-lowering activity, a prior report shows that statins inhibit mTORC1 signaling through protein prenylation, at least in coronary arterial myocytes (24). Thus, condition-dependent mechanisms may exist.

Our results fundamentally differ from a previous report proposing the involvement of geranylgeranyl biosynthesis in statin-mediated induction of autophagy during *M. tuberculosis* infection (9). Our conclusions are strongly supported by the findings that the anti-mycobacterial activity of statins is phenocopied only by cholesterol-branch but not by prenylation-branch inhibitors and the biological effects of simvastatin on the AMPK-mTORC1-TFEB-autophagy axis are reversed by adding exogenous cholesterol to the cells. We propose that the different conclusions reached by the previous authors most likely depend on their employment of murine cells and very high simvastatin doses (500-fold higher than the dose we used), which cannot distinguish between anti-cholesterol and anti-prenylation effects of the statin, are toxic for human (THP1) cells, and far exceed the plasma levels attained during therapeutic administration of simvastatin (72).

Our work shows that the cholesterol-lowering activity of statins can affect autophagy in multiple interconnected ways. First, statins induce AMPK signaling by increasing expression of LKB1 and AMPK genes and the ratio of intracellular AMP:ATP, which activates AMPK (73). The effect on the AMP:ATP ratio is presumably achieved by impairing mitochondrial function, as previously shown (64). Second, statins inhibit mTORC1 activation by decreasing cholesterol biosynthesis and altering homeostasis of cellular cholesterol, as we discussed above. Third, mTORC1 inhibition may also occur by activating AMPK signaling, which prevents mTORC1 activation (74). In addition, a double-negative feedback loop between mTORC1 and AMPK has recently been proposed (75). Statins fully counter the effects of *M. tuberculosis* infection on the key AMPK-mTORC1-autophagy axis (Fig. 7), which strongly supports the potential value of this drug class as anti-tubercular therapeutics.

A role for TFEB in the anti-tubercular activity of statins, as demonstrated in the present work, implies that TFEB may be classified as a target for anti-tuberculosis therapeutic intervention. Because TFEB directly regulates autophagy-inducing effector functions, compounds targeting TFEB may exhibit fewer pleiotropic effects, and presumably fewer unwanted side effects, than those targeting upstream factors like mTORC1. Indeed, everolimus, a rapamycin-analog (76) that is under consideration for use in host-directed therapy against tuberculosis (77), may cause immunosuppression (78) and facilitate reactivation of latent *M. tuberculosis* infection (79). Thus, further investigation will be needed to determine the therapeutic dose of everolimus and the optimal mode of its delivery. The work presented here not only provides the molecular basis for the anti-tubercular activity of statins, but also fosters investigations into novel therapeutic strategies to combat tuberculosis.

The mTORC1-AMPK-TFEB axis has emerged as a key lysosome-based regulator of many biological processes, including autophagy, cellular metabolism, immune responses, inflammation, and damage resolution (80–82). In addition, this axis has a functional impact on host-pathogen responses. For example, AMPK activation is beneficial in infections with hepatitis B and C viruses, but it is detrimental with Dengue and Ebola viruses (83). In another example, *Leishmania major* produces proteases that block mTOR activation, thus repressing the type 1 interferon response and enabling the parasite to survive inside cells (84). *Listeria monocytogenes* and *Staphylococcus aureus* stimulate mTOR by promoting activation of phosphoinositide 3-kinase/protein kinase B signaling pathways, which favor pathogen survival during infection (85). The changed perspective on statin-mediated modulation of the mTORC1-AMPK-TFEB axis resulting from the present work will spur novel knowledge-based strategies of host-directed therapies against various infectious agents.

### Data availability

The data that support the findings of this study are all listed in the article and available from the corresponding author upon reasonable request.

## Supplementary Material

Supplemental Data
