# Supplementary material for: The anti-tubercular activity of simvastatin is mediated by cholesterol-driven autophagy via the AMPK-mTORC1-TFEB axis
Source: J Lipid Res. 2020 Aug 26;61(12):1617–28. doi: 10.1194/jlr.RA120000895 (PMC7707180; doi:10.1194/jlr.RA120000895)
Supplement: Supplemental Data [file supp_61_12_1617__index.html]

The anti-tubercular activity of simvastatin is mediated by cholesterol-driven autophagy via the AMPK-mTORC1-TFEB axis. — Simvastatin induces autophagy via AMPK-mTORC1-TFEB axis — The anti-tubercular activity of simvastatin is mediated by cholesterol-driven autophagy via the AMPK-mTORC1-TFEB axis — Supplemental Data 

# The anti-tubercular activity of simvastatin is mediated by cholesterol-driven autophagy via the AMPK-mTORC1-TFEB axis

## Supplemental Data

- The anti-tubercular activity of simvastatin is mediated by cholesterol-driven autophagy via the AMPK-mTORC1-TFEB axis. - Supplemental material
