# Supplementary material for: The anti-tubercular activity of simvastatin is mediated by cholesterol-driven autophagy via the AMPK-mTORC1-TFEB axis
Source: J Lipid Res. 2020 Aug 26;61(12):1617–28. doi: 10.1194/jlr.RA120000895 (PMC7707180; doi:10.1194/jlr.RA120000895)
Supplement: Supplemental Data [file supp_RA120000895_160771_2_supp_585639_qfprd3.docx]

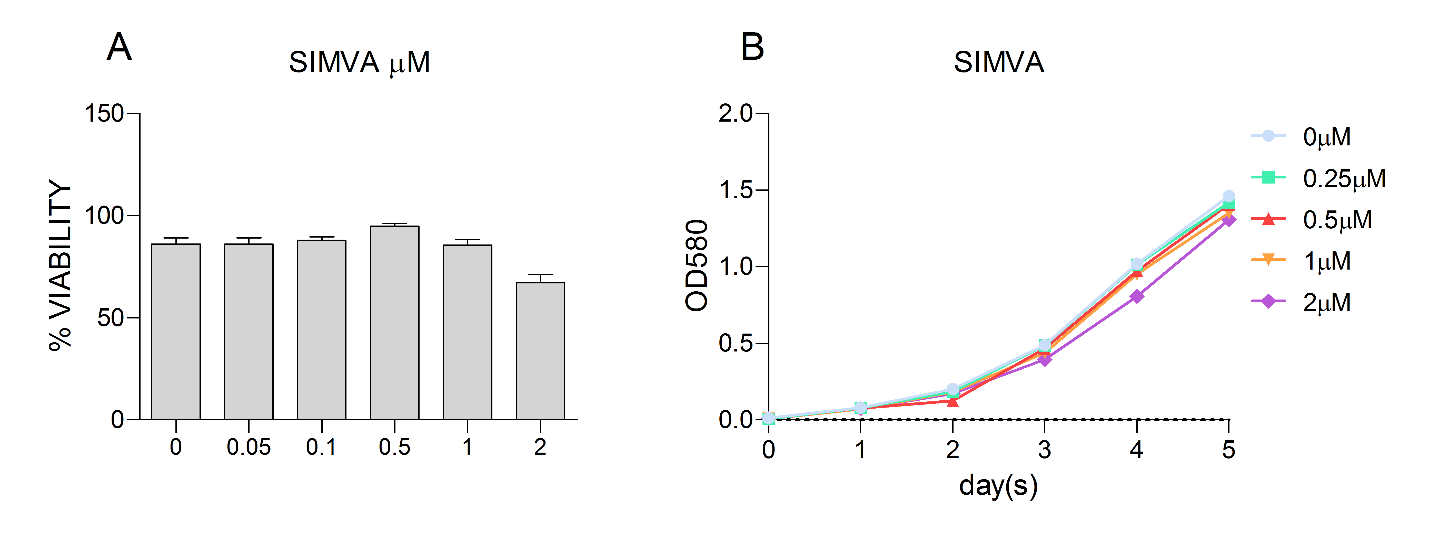


**Supplemental Figure S1. Effect of simvastatin on *M. tuberculosis* axenic cultures and toxicity of THP1 cells** (**A**) *M. tuberculosis* at an optical density (OD) 580 of 0.01 was exposed to simvastatin at the indicated doses for 5 days. Growth of the bacilli was recorded by using a spectrophotometer. (**B**) Cell viability of infected THP1 cells treated with simvastatin at the indicated doses for 6 days calculated was evaluated using trypan blue exclusion (viability was affected at 2µM).


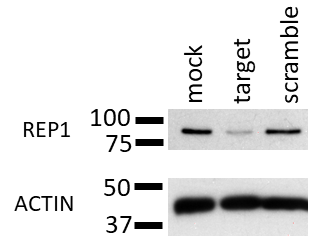


**Supplemental Figure S2. siRNA-mediated protein knockdown.** Western blot analysis of REP-1 and beta-actin. Lane 1: mock transfected THP1 cells with transfection reagent, lane 2: THP1 cells transfected with targeting siRNA, and lane 3: THP1 cells transfected with non-targeting siRNA.


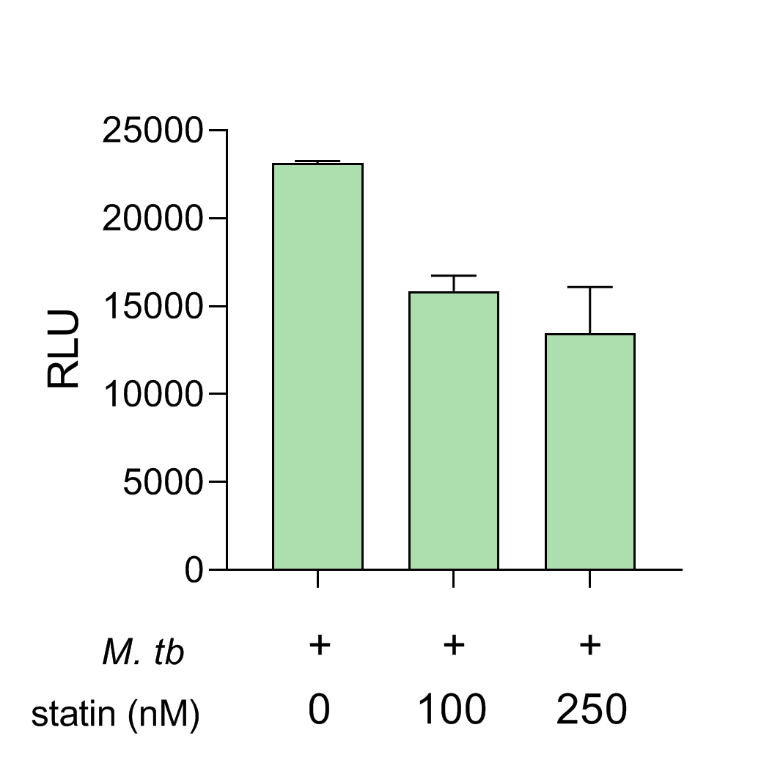


**Supplemental Figure S3.** **Reduction of *M. tuberculosis* growth by simvastatin.** Effect of 100 and 250 nM simvastatin on the intracellular growth of *M. tuberculosis* in THP1 cells. Since the strain expresses the *lux* operon [1], growth was monitored using a luminometer and recorded as relative light units (RLU).

**Supplemental Table S1.** Calculated p-values were determined using the one-sided T-test to compare transcript levels in the mTOR signaling pathway (KEGG Pathways (2017-04-21)) between simvastatin treatment (12 hrs) and solvent control. P-values for multiple transcripts for each gene measured on the microarray platform were first integrated in a nested CERNO test and sorted as described in Methods.

| **Gene** | **GeneID** | **MinProbeP** | **MinProbeP_BH** |
| --- | --- | --- | --- |
| GRB10 | [2887](http://www.ncbi.nlm.nih.gov/gene/2887) | 0.001109824 | 0.430328378 |
| ULK2 | [9706](http://www.ncbi.nlm.nih.gov/gene/9706) | 0.000932144 | 0.421150989 |
| SESN2 | [83667](http://www.ncbi.nlm.nih.gov/gene/83667) | 0.001436966 | 0.443984358 |
| IGF1R | [3480](http://www.ncbi.nlm.nih.gov/gene/3480) | 0.004732249 | 0.450956278 |
| EIF4EBP1 | [1978](http://www.ncbi.nlm.nih.gov/gene/1978) | 0.001242791 | 0.443984358 |
| INSR | [3643](http://www.ncbi.nlm.nih.gov/gene/3643) | 0.013018491 | 0.47852084 |
| BRAF | [673](http://www.ncbi.nlm.nih.gov/gene/673) | 0.001555621 | 0.443984358 |
| SLC7A5 | [8140](http://www.ncbi.nlm.nih.gov/gene/8140) | 0.002160195 | 0.443984358 |
| RPS6 | [6194](http://www.ncbi.nlm.nih.gov/gene/6194) | 0.017128786 | 0.483459095 |
| PTEN | [5728](http://www.ncbi.nlm.nih.gov/gene/5728) | 0.011421643 | 0.47852084 |
| FZD7 | [8324](http://www.ncbi.nlm.nih.gov/gene/8324) | 0.015526197 | 0.482266701 |
| KRAS | [3845](http://www.ncbi.nlm.nih.gov/gene/3845) | 0.001753226 | 0.443984358 |
| ULK1 | [8408](http://www.ncbi.nlm.nih.gov/gene/8408) | 0.006171159 | 0.469816424 |
| MLST8 | [64223](http://www.ncbi.nlm.nih.gov/gene/64223) | 0.00629089 | 0.469816424 |
| AKT3 | [10000](http://www.ncbi.nlm.nih.gov/gene/10000) | 0.001886011 | 0.443984358 |
| PRKCB | [5579](http://www.ncbi.nlm.nih.gov/gene/5579) | 0.025957045 | 0.497540389 |
| RPS6KA2 | [6196](http://www.ncbi.nlm.nih.gov/gene/6196) | 0.026284887 | 0.498394638 |
| EIF4E2 | [9470](http://www.ncbi.nlm.nih.gov/gene/9470) | 0.013837528 | 0.47852084 |
| MAPKAP1 | [79109](http://www.ncbi.nlm.nih.gov/gene/79109) | 0.025432697 | 0.495000508 |
| RRAGD | [58528](http://www.ncbi.nlm.nih.gov/gene/58528) | 0.023325731 | 0.491452839 |
| AKT2 | [208](http://www.ncbi.nlm.nih.gov/gene/208) | 0.009170944 | 0.469816424 |
| STRADB | [55437](http://www.ncbi.nlm.nih.gov/gene/55437) | 0.017106803 | 0.483459095 |
| DDIT4 | [54541](http://www.ncbi.nlm.nih.gov/gene/54541) | 0.017529493 | 0.484205568 |
| RRAGB | [10325](http://www.ncbi.nlm.nih.gov/gene/10325) | 0.018359747 | 0.485255556 |
| SLC38A9 | [153129](http://www.ncbi.nlm.nih.gov/gene/153129) | 0.034598298 | 0.51335088 |
| TNFRSF1A | [7132](http://www.ncbi.nlm.nih.gov/gene/7132) | 0.019111025 | 0.485255556 |
| MAPK3 | [5595](http://www.ncbi.nlm.nih.gov/gene/5595) | 0.021819564 | 0.489762465 |
| PIK3R1 | [5295](http://www.ncbi.nlm.nih.gov/gene/5295) | 0.0407176 | 0.522684349 |
| RRAGC | [64121](http://www.ncbi.nlm.nih.gov/gene/64121) | 0.048074372 | 0.533371235 |
| LAMTOR1 | [55004](http://www.ncbi.nlm.nih.gov/gene/55004) | 0.033429789 | 0.512020729 |
| SOS2 | [6655](http://www.ncbi.nlm.nih.gov/gene/6655) | 0.030070733 | 0.506764922 |
| DEPTOR | [64798](http://www.ncbi.nlm.nih.gov/gene/64798) | 0.012097783 | 0.47852084 |
| EIF4B | [1975](http://www.ncbi.nlm.nih.gov/gene/1975) | 0.033837131 | 0.5122758 |
| LAMTOR2 | [28956](http://www.ncbi.nlm.nih.gov/gene/28956) | 0.060804675 | 0.55276242 |
| MAP2K2 | [5605](http://www.ncbi.nlm.nih.gov/gene/5605) | 0.074739787 | 0.574982329 |
| NPRL2 | [10641](http://www.ncbi.nlm.nih.gov/gene/10641) | 0.043228173 | 0.524298087 |
| WNT6 | [7475](http://www.ncbi.nlm.nih.gov/gene/7475) | 0.031691656 | 0.511284177 |
| WDR59 | [79726](http://www.ncbi.nlm.nih.gov/gene/79726) | 0.028230583 | 0.503194159 |
| FZD1 | [8321](http://www.ncbi.nlm.nih.gov/gene/8321) | 0.075315677 | 0.5755359 |
| DVL2 | [1856](http://www.ncbi.nlm.nih.gov/gene/1856) | 0.088263221 | 0.581068377 |
| WNT2B | [7482](http://www.ncbi.nlm.nih.gov/gene/7482) | 0.021182142 | 0.488465265 |
| ATP6V1E2 | [90423](http://www.ncbi.nlm.nih.gov/gene/90423) | 0.11925257 | 0.593878314 |
| TSC2 | [7249](http://www.ncbi.nlm.nih.gov/gene/7249) | 0.127442843 | 0.599279544 |
| DEPDC5 | [9681](http://www.ncbi.nlm.nih.gov/gene/9681) | 0.137480168 | 0.610375159 |
| FNIP1 | [96459](http://www.ncbi.nlm.nih.gov/gene/96459) | 0.01838442 | 0.485255556 |
| WNT5A | [7474](http://www.ncbi.nlm.nih.gov/gene/7474) | 0.051121338 | 0.538393653 |
| WNT10A | [80326](http://www.ncbi.nlm.nih.gov/gene/80326) | 0.117448245 | 0.593115239 |
| WNT8B | [7479](http://www.ncbi.nlm.nih.gov/gene/7479) | 0.15086932 | 0.624017139 |
| RPS6KA6 | [27330](http://www.ncbi.nlm.nih.gov/gene/27330) | 0.070306664 | 0.568531923 |
| RNF152 | [220441](http://www.ncbi.nlm.nih.gov/gene/220441) | 0.080750723 | 0.581068377 |
| LAMTOR3 | [8649](http://www.ncbi.nlm.nih.gov/gene/8649) | 0.153425297 | 0.626745609 |
| FZD5 | [7855](http://www.ncbi.nlm.nih.gov/gene/7855) | 0.06031968 | 0.551298914 |
| SOS1 | [6654](http://www.ncbi.nlm.nih.gov/gene/6654) | 0.049490311 | 0.535249151 |
| NRAS | [4893](http://www.ncbi.nlm.nih.gov/gene/4893) | 0.097327924 | 0.581068377 |
| SLC3A2 | [6520](http://www.ncbi.nlm.nih.gov/gene/6520) | 0.189841661 | 0.662664415 |
| MAP2K1 | [5604](http://www.ncbi.nlm.nih.gov/gene/5604) | 0.191960298 | 0.664760673 |
| WDR24 | [84219](http://www.ncbi.nlm.nih.gov/gene/84219) | 0.203396803 | 0.676407272 |
| WNT9B | [7484](http://www.ncbi.nlm.nih.gov/gene/7484) | 0.210123965 | 0.67906154 |
| IRS1 | [3667](http://www.ncbi.nlm.nih.gov/gene/3667) | 0.096786186 | 0.581068377 |
| RPS6KA1 | [6195](http://www.ncbi.nlm.nih.gov/gene/6195) | 0.214463557 | 0.681974213 |
| FZD2 | [2535](http://www.ncbi.nlm.nih.gov/gene/2535) | 0.165364898 | 0.638522599 |
| PIK3R2 | [5296](http://www.ncbi.nlm.nih.gov/gene/5296) | 0.100054089 | 0.581386593 |
| ATP6V1C2 | [245973](http://www.ncbi.nlm.nih.gov/gene/245973) | 0.148829059 | 0.621413037 |
| AKT1S1 | [84335](http://www.ncbi.nlm.nih.gov/gene/84335) | 0.20429009 | 0.677398581 |
| WNT1 | [7471](http://www.ncbi.nlm.nih.gov/gene/7471) | 0.274083847 | 0.7378545 |
| NPRL3 | [8131](http://www.ncbi.nlm.nih.gov/gene/8131) | 0.16724259 | 0.640306024 |
| FLCN | [201163](http://www.ncbi.nlm.nih.gov/gene/201163) | 0.150405227 | 0.623509226 |
| TBC1D7 | [51256](http://www.ncbi.nlm.nih.gov/gene/51256) | 0.159742801 | 0.632967078 |
| FZD4 | [8322](http://www.ncbi.nlm.nih.gov/gene/8322) | 0.12268478 | 0.595148053 |
| PRKCG | [5582](http://www.ncbi.nlm.nih.gov/gene/5582) | 0.175996141 | 0.649562782 |
| WNT4 | [54361](http://www.ncbi.nlm.nih.gov/gene/54361) | 0.118067005 | 0.593115239 |
| WNT2 | [7472](http://www.ncbi.nlm.nih.gov/gene/7472) | 0.320296942 | 0.776474926 |
| FZD10 | [11211](http://www.ncbi.nlm.nih.gov/gene/11211) | 0.325652817 | 0.780762987 |
| CHUK | [1147](http://www.ncbi.nlm.nih.gov/gene/1147) | 0.338654657 | 0.791085931 |
| RHEB | [6009](http://www.ncbi.nlm.nih.gov/gene/6009) | 0.079515595 | 0.581068377 |
| PIK3CA | [5290](http://www.ncbi.nlm.nih.gov/gene/5290) | 0.072933318 | 0.572924748 |
| WNT5B | [81029](http://www.ncbi.nlm.nih.gov/gene/81029) | 0.120101513 | 0.59429342 |
| GSK3B | [2932](http://www.ncbi.nlm.nih.gov/gene/2932) | 0.070470671 | 0.568595442 |
| WNT7A | [7476](http://www.ncbi.nlm.nih.gov/gene/7476) | 0.392161441 | 0.831620814 |
| RHOA | [387](http://www.ncbi.nlm.nih.gov/gene/387) | 0.188268426 | 0.66052823 |
| SEH1L | [81929](http://www.ncbi.nlm.nih.gov/gene/81929) | 0.281114595 | 0.74463589 |
| CLIP1 | [6249](http://www.ncbi.nlm.nih.gov/gene/6249) | 0.069498256 | 0.568531923 |
| ATP6V1C1 | [528](http://www.ncbi.nlm.nih.gov/gene/528) | 0.072178463 | 0.570914588 |
| RRAGA | [10670](http://www.ncbi.nlm.nih.gov/gene/10670) | 0.440891437 | 0.867013879 |
| ATP6V1D | [51382](http://www.ncbi.nlm.nih.gov/gene/51382) | 0.099906682 | 0.581386593 |
| RPS6KB2 | [6199](http://www.ncbi.nlm.nih.gov/gene/6199) | 0.476173439 | 0.889098517 |
| INS | [3630](http://www.ncbi.nlm.nih.gov/gene/3630) | 0.477696635 | 0.889894726 |
| LRP5 | [4041](http://www.ncbi.nlm.nih.gov/gene/4041) | 0.278231083 | 0.741804826 |
| MAPK1 | [5594](http://www.ncbi.nlm.nih.gov/gene/5594) | 0.072083034 | 0.570555939 |
| WNT11 | [7481](http://www.ncbi.nlm.nih.gov/gene/7481) | 0.532316263 | 0.919227738 |
| ATP6V1B2 | [526](http://www.ncbi.nlm.nih.gov/gene/526) | 0.536598735 | 0.92153058 |
| SGK1 | [6446](http://www.ncbi.nlm.nih.gov/gene/6446) | 0.542085215 | 0.923699015 |
| WNT8A | [7478](http://www.ncbi.nlm.nih.gov/gene/7478) | 0.544991102 | 0.925230986 |
| DVL3 | [1857](http://www.ncbi.nlm.nih.gov/gene/1857) | 0.291089352 | 0.752366244 |
| AKT1 | [207](http://www.ncbi.nlm.nih.gov/gene/207) | 0.552424577 | 0.928954124 |
| WNT10B | [7480](http://www.ncbi.nlm.nih.gov/gene/7480) | 0.569621293 | 0.9383551 |
| FZD9 | [8326](http://www.ncbi.nlm.nih.gov/gene/8326) | 0.583207883 | 0.94466096 |
| ATP6V1B1 | [525](http://www.ncbi.nlm.nih.gov/gene/525) | 0.290341236 | 0.751854121 |
| PDPK1 | [5170](http://www.ncbi.nlm.nih.gov/gene/5170) | 0.611189618 | 0.954444297 |
| IKBKB | [3551](http://www.ncbi.nlm.nih.gov/gene/3551) | 0.289651865 | 0.751029291 |
| TSC1 | [7248](http://www.ncbi.nlm.nih.gov/gene/7248) | 0.623227067 | 0.960129794 |
| PIK3CB | [5291](http://www.ncbi.nlm.nih.gov/gene/5291) | 0.373978832 | 0.818307275 |
| STK11 | [6794](http://www.ncbi.nlm.nih.gov/gene/6794) | 0.199244725 | 0.672372544 |
| WNT16 | [51384](http://www.ncbi.nlm.nih.gov/gene/51384) | 0.418934473 | 0.851750995 |
| PRKCA | [5578](http://www.ncbi.nlm.nih.gov/gene/5578) | 0.129900376 | 0.601143843 |
| GRB2 | [2885](http://www.ncbi.nlm.nih.gov/gene/2885) | 0.125690656 | 0.598327418 |
| HRAS | [3265](http://www.ncbi.nlm.nih.gov/gene/3265) | 0.695605065 | 0.986581513 |
| MTOR | [2475](http://www.ncbi.nlm.nih.gov/gene/2475) | 0.354796062 | 0.803898789 |
| SKP2 | [6502](http://www.ncbi.nlm.nih.gov/gene/6502) | 0.243846648 | 0.711144578 |
| ATP6V1G3 | [127124](http://www.ncbi.nlm.nih.gov/gene/127124) | 0.719031911 | 0.993979891 |
| ATP6V1G2 | [534](http://www.ncbi.nlm.nih.gov/gene/534) | 0.730911414 | 0.997595288 |
| RAF1 | [5894](http://www.ncbi.nlm.nih.gov/gene/5894) | 0.305262064 | 0.764579102 |
| WNT7B | [7477](http://www.ncbi.nlm.nih.gov/gene/7477) | 0.365826655 | 0.811839376 |
| CAB39 | [51719](http://www.ncbi.nlm.nih.gov/gene/51719) | 0.518241681 | 0.911390542 |
| LRP6 | [4040](http://www.ncbi.nlm.nih.gov/gene/4040) | 0.410719583 | 0.845363548 |
| DVL1 | [1855](http://www.ncbi.nlm.nih.gov/gene/1855) | 0.822491981 | 0.999996506 |
| SEC13 | [6396](http://www.ncbi.nlm.nih.gov/gene/6396) | 0.832058292 | 0.999996506 |
| LAMTOR4 | [389541](http://www.ncbi.nlm.nih.gov/gene/389541) | 0.839767875 | 0.999996506 |
| RPS6KA3 | [6197](http://www.ncbi.nlm.nih.gov/gene/6197) | 0.561248385 | 0.933500843 |
| RPS6KB1 | [6198](http://www.ncbi.nlm.nih.gov/gene/6198) | 0.463941937 | 0.881512253 |
| CAB39L | [81617](http://www.ncbi.nlm.nih.gov/gene/81617) | 0.23092078 | 0.698891595 |
| LAMTOR5 | [10542](http://www.ncbi.nlm.nih.gov/gene/10542) | 0.699713516 | 0.988252244 |
| IGF1 | [3479](http://www.ncbi.nlm.nih.gov/gene/3479) | 0.29278256 | 0.753931023 |
| TELO2 | [9894](http://www.ncbi.nlm.nih.gov/gene/9894) | 0.725863069 | 0.996436457 |
| WNT9A | [7483](http://www.ncbi.nlm.nih.gov/gene/7483) | 0.709192336 | 0.990926278 |
| ATP6V1E1 | [529](http://www.ncbi.nlm.nih.gov/gene/529) | 0.942185572 | 0.999996506 |
| ATP6V1G1 | [9550](http://www.ncbi.nlm.nih.gov/gene/9550) | 0.811289889 | 0.999996506 |
| RPTOR | [57521](http://www.ncbi.nlm.nih.gov/gene/57521) | 0.955653562 | 0.999996506 |
| TTI1 | [9675](http://www.ncbi.nlm.nih.gov/gene/9675) | 0.961885455 | 0.999996506 |
| MIOS | [54468](http://www.ncbi.nlm.nih.gov/gene/54468) | 0.346511001 | 0.797425998 |
| EIF4E | [1977](http://www.ncbi.nlm.nih.gov/gene/1977) | 0.341168316 | 0.793392859 |
| STRADA | [92335](http://www.ncbi.nlm.nih.gov/gene/92335) | 0.67751961 | 0.978906917 |
| PRKAA2 | [5563](http://www.ncbi.nlm.nih.gov/gene/5563) | 0.377930069 | 0.820872939 |
| ATP6V1H | [51606](http://www.ncbi.nlm.nih.gov/gene/51606) | 0.455557934 | 0.876178968 |
| ATP6V1A | [523](http://www.ncbi.nlm.nih.gov/gene/523) | 0.847559077 | 0.999996506 |
| FZD6 | [8323](http://www.ncbi.nlm.nih.gov/gene/8323) | 0.981719961 | 0.999996506 |
| FZD3 | [7976](http://www.ncbi.nlm.nih.gov/gene/7976) | 0.592488057 | 0.948091877 |
| RICTOR | [253260](http://www.ncbi.nlm.nih.gov/gene/253260) | 0.473910736 | 0.887761008 |
| PIK3CD | [5293](http://www.ncbi.nlm.nih.gov/gene/5293) | 0.831190851 | 0.999996506 |
| PIK3R3 | [8503](http://www.ncbi.nlm.nih.gov/gene/8503) | 0.907976527 | 0.999996506 |
| PRR5 | [55615](http://www.ncbi.nlm.nih.gov/gene/55615) | 0.918227678 | 0.999996506 |
| TNF | [7124](http://www.ncbi.nlm.nih.gov/gene/7124) | 0.996733204 | 0.999996506 |
| FNIP2 | [57600](http://www.ncbi.nlm.nih.gov/gene/57600) | 0.576956264 | 0.941554496 |
| PRKAA1 | [5562](http://www.ncbi.nlm.nih.gov/gene/5562) | 0.471421016 | 0.886175554 |
| LPIN1 | [23175](http://www.ncbi.nlm.nih.gov/gene/23175) | 0.957125372 | 0.999996506 |

**Reference**

1. Dutta NK, Bruiners N, Pinn ML, Zimmerman MD, Prideaux B, Dartois V, Gennaro ML, Karakousis PC. Statin adjunctive therapy shortens the duration of TB treatment in mice. The Journal of antimicrobial chemotherapy. 2016;71(6):1570-7, 10.1093/jac/dkw014.
